# The Value of Detecting Creatinine and Human Chorionic Gonadotropin (Hcg) Levels in Cervicovaginal Discharge in Identifying Leakage in Pregnant Women 24 to 37 Weeks with Clear Leakage or Positive Fern Test: Value of Detecting Creatinine and Hcg Levels in Cervicovaginal Discharge in Identifying Leakage in Pregnant Women

**DOI:** 10.31661/gmj.v14i.3693

**Published:** 2025-03-17

**Authors:** Sepideh Miraj, Abolfazl Mohammadbeigi, Maryam Hashemi Kazeroni

**Affiliations:** ^1^ Department of Obstetrics and Gynecology, School of Medicine, Nekouei-Hedayati-Forghani Hospital, Qom University of Medical Sciences, Qom, Iran; ^2^ Department of Biostatistics and Epidemiology, School of Health, Research Center for Environmental Pollutants, Qom University of Medical Sciences, Qom, Iran

**Keywords:** Preterm Rupture of Membranes (PROM), Creatinine, Human Chorionic Gonadotropin (HCG)

## Abstract

**Background:**

Premature birth is one of the most important midwifery problems, and its early diagnosis is essential in the treatment and prevention process. Therefore, the purpose of this study is the value of detecting the level of creatinine and human chorionic gonadotropin (HCG) in cervicovaginal secretions in detecting discharge in pregnant women of 24 to 37 weeks with clear discharge or a positive Fern test in Forghani Hospital in 2022.

**Materials and Methods:**

This study was conducted on 230 pregnant women from 24 to 37 weeks suspected of having vaginal discharge. In all suspected women, the creatinine and HCG levels of cervicovaginal secretions were measured, and the results were analyzed based on clear discharge and Fern’s test, then the diagnostic power was calculated based on Rock’s curve.

**Results:**

Our study’s findings showed that the average levels of creatinine and B-HCG in women with clear discharge or a positive Fern test were significantly higher than in other women. However, by examining the diagnostic accuracy, it was found that the degree of agreement between the two tests in diagnosing discharge Clear and positive Fern test is weak to moderate, and creatinine has high sensitivity and B-HCG has high specificity.

**Conclusion:**

Creatinine and BHCG can be used in the diagnosis of premature rupture along with standard tests, and due to the use of a valuable diagnostic method, the fatal complications of PROM can be prevented, and with the timely treatment of this complication, the dangerous consequences are reduced, a study with higher sample volume and careful control of confounders is required.

## Introduction

Preterm labor, which occurs before 37 weeks of gestation, accounts for 5 to 15 percent (on average 10 percent) of all deliveries. Despite significant advancements in the treatment of this adverse pregnancy outcome, its prevalence has not decreased over the past two decades [1]. The complications of preterm labor include intraventricular hemorrhage in newborns, an increased rate of cesarean deliveries, cerebral palsy, neurological complications, very high costs, and other associated issues [2]. In preterm pregnancies, the rupture of the amniotic membrane results in the early onset of labor, rendering any efforts to prevent preterm delivery ineffective [3]. Rupture of the fetal membranes before delivery is a notable complication in obstetrics. This condition occurs in roughly 10% of all pregnancies. Approximately 25% of cases of premature rupture of membranes (PROM) are categorized as very preterm, leading to the birth of extremely preterm infants [4]. In situations where the rupture or leakage of amniotic fluid is minimal, diagnosing the condition can be difficult. An accurate diagnosis of amniotic membrane rupture is crucial, particularly in cases like preterm pregnancies, where membrane rupture may lead to premature labor, rendering any attempts to prevent preterm delivery ineffective [5]. Appropriate treatment and timely intervention require a precise and rapid diagnostic method. In term pregnancies, a long interval between membrane rupture and delivery is associated with an increased incidence of intrauterine infection [6]. Maternal mortality and neonatal complications increase after rupture of the amniotic sac. Perinatal mortality rates typically rise 2 and 4 times the usual levels within 24 and 48 hours, respectively, following the rupture [7]. Several methods have been suggested for diagnosing amniotic sac rupture, each varying in diagnostic accuracy [8]. Given the time needed to obtain results and make informed decisions regarding appropriate treatment, these methods are consistently valuable. In a typical case, the diagnosis of amniotic sac rupture is based on the patient’s report and the observation of amniotic fluid from the cervix. The presence of amniotic fluid in the vagina after the insertion of a speculum confirms the diagnosis. There are cases where amniotic fluid is not visible in the speculum, yet the patient reports a history of pooling amniotic fluid and some time has passed since the rupture. Additionally, the alkalinity test of cervical secretions using nitrazine (nitrazine test) and the examination of crystallization of cervical secretions on a slide (Fern test) is performed [9][10]. The nitrazine test may be falsely negative after 48 hours. Additionally, the presence of semen, blood, cervicitis, vaginitis, urinary contamination, and the use of antiseptics can increase the likelihood of a false negative result [11]. The rate of false-positive results for the Fern test is between 5-30%. The main issue with the Fern test is the difficulty in diagnosing cases where a significant amount of time has passed since the rupture of the amniotic sac, or when there is a presence of blood secretions or a large amount of infection. In these cases, the result may be a false positive. Measuring the amniotic fluid index via ultrasound, while useful, is not reliable as it cannot differentiate oligohydramnios from other causes versus fluid reduction due to rupture and leakage. Consequently, it has a high rate of false positives and negatives [12]. The Amnisure test represents the newest approach for diagnosing membrane rupture. This test identifies trace amounts of placenta alpha-1 microglobulin in vaginal secretions. This protein is present in high concentrations (200-25000 ng/ml) in amniotic fluid, while it exists in low levels in the blood and minimal amounts in vaginal secretions. The sensitivity of this test for detecting PAMG-1 ranges from .005 to .05 ml, allowing it to identify even subclinical cases in which clear amniotic fluid leakage is not observed. Several studies have been conducted on biochemical factors with high concentrations in amniotic fluid. Prolactin, alpha-fetoprotein, insulin-like growth factor (IGF), fibronectin, and human chorionic gonadotropin beta (HCGβ), as well as placental alpha-microglobulin, are among these factors [13]. Recently, the use of urea, creatinine, and BHCG found in vaginal fluid has been identified as predictive factors for diagnosing PROM (premature rupture of membranes [14][15]. The study aims to find a simple, cost-effective, and accessible method for assessing leakage in pregnant women with high sensitivity and specificity, as well as to determine the cutoff point by evaluating creatinine and BHCG in the cervical and vaginal secretions of pregnant women.

## Materials and Methods

This study was conducted using a cross-sectional analytical method. The study population included pregnant women between 24 to 37 weeks of gestation who presented to the obstetric emergency department of Forghani Hospital in 2022 with a complaint of vaginal leakage. The sample size was calculated to be 230 individuals, with a power of 80% and a margin of difference of 10% (α = 0.05, β = 0.2), based on the results of a similar study. Sampling was also conducted using a convenient sampling method. The inclusion criteria included: gestational age of 24 to 37 weeks, vaginal leakage, no underlying diseases, and no obstetric complications. The exclusion criteria included: any vaginal bleeding (such as previa, abruption, or traumatic), exposure of the sample to urine, intercourse the night before, use of vaginal cleansers, obvious vaginal infections, the presence of fetal anomalies, preeclampsia, and hypertension due to pregnancy, intrauterine growth restriction (IUGR), and intrauterine fetal demise. After obtaining informed consent from the patients, data including menstrual history, obstetric history, presenting complaints, general examination, abdominal examination, and speculum examination were collected and recorded. The patient was positioned in a lithotomy position with appropriate lighting. A sterile vaginal examination was performed using a sterile speculum. In patients, vaginal fluid was directly aspirated from the posterior fornix using a sterile 5 ml syringe, and the samples were collected in plain vials, which were stored at a temperature of 4 to 8 degrees Celsius and transferred to the laboratory in the pathology department for the estimation of β-hCG levels in the vaginal fluid. The sample was centrifuged at 2500 revolutions per minute for 5 minutes, and the β-hCG titer was determined using the Chemiluminescent Microparticle Immunoassay (CMIA) method. The total duration of the assay was 15 to 20 minutes. To determine β-hCG and creatinine levels, the patient was positioned in a supine lithotomy position, and a speculum was inserted. Then, 5 cc of sterile water was injected into the posterior cul-de-sac using a syringe. After a few minutes, 3 cc of this fluid was aspirated into the syringe and transferred into test tubes. The samples were then transported to the hospital’s pathology laboratory while maintaining a cold chain. After centrifugation and debris separation, the samples were evaluated for β-hCG and creatinine levels. Creatinine was measured using the auto analyzer method with the Prestige autoanalyzer, while β-hCG was quantified using a quantitative luminescence method with the Abbott device from the USA. Then, the data were entered into IBM SPSS Statistics for Windows, version 26 (IBM Corp., Armonk, N.Y., USA), and the sensitivity, specificity, positive predictive value, negative predictive value, and cutoff points for creatinine and β-hCG levels in cervical-vaginal secretions were calculated using ROC (Receiver Operating Characteristic) curves.

## Results

**Table:** Table1. Examining the studied variables in women suspected of discharge

Variable	Mean	SD
Age (year)	29.18	5.63
Weight (Kg)	72.6	6.68
GA (week)	34.6	3.2
Gravid	1.98	1.19
Para	0.68	0.87
(mg/dl)Cr	0.36	0.43
(mIU/ml ) B-HCG	229.03	549.7

**Table:** Table2. Comparison of Demographic and Laboratory Variables Between Women with and without Suspicious Fluid Leakage

Variable	Number	Mean	SD	P-value
Age (year)	176	29.5	5.6	0.067
	54	27.9	5.3	
Weight (Kg)	176	72.64	6.94	0.953
	54	72.7	5.77	
GA (week)	176	34.54	3.34	0.497
	54	34.87	3.04	
Gravid	176	1.98	1.21	0.911
	54	1.96	1.13	
Para	176	0.72	0.84	0.259
	54	0.56	0.96	
(mg/dl) Cr	176	0.25	0.28	<0.001
	54	0.77	0.55	
B-HCG (mIU/ml )	176	123.57	312.91	<0.001
	54	572.76	908.41	

**Table:** Table3. Comparison of Demographic and Laboratory Variables Between Women with Positive and Negative Fern Tests

Variable	Fern Tests	Number	Mean	SD	P-value
Age (year)	Negative	149	29.49	5.82	0.241
	Positive	81	28.6	5.23	
Weight	Negative	149	72.56	7.13	0.776
	Positive	81	72.83	5.79	
GA (week)	Negative	149	34.4	3.42	0.254
	Positive	81	34.94	2.97	
Gravidity	Negative	149	1.94	1.17	0.513
	Positive	81	2.05	1.23	
Parity	Negative	149	0.73	0.82	0.347
	Positive	81	0.6	0.971	
(mg/dl)Cr	Negative	149	0.2	0.233	<0.001
	Positive	81	0.68	0.528	
(mIU/ml ) B-HCG	Negative	149	61.13	104.96	<0.001
	Positive	81	537.9	834.04	

**Table:** Table4. The area under the ROC curve (AUC) for creatinine and B-HCG in the diagnosis of suspected leakage in women

Variable	AUC	Standard ad Error	Sig	Confidence interval
				Minimum	Maximum
Cr	0.833	0.029	0.000	0.776	0.889
B-HCG	0.672	0.043	0.000	0.582	0.757

**Table:** Table5. The sensitivity and specificity of creatinine and B-HCG in diagnosing fluid leakage in women under study based on the optimal cutoff point

	Cut-off Point	Sensitivity	Specificity	PPV	NPV	LR-	LR+	Overall Accuracy	Kappa	Significance
Cr	0.15	94.4 %	58 %	40.8 %	97.1 %	0.096	2.24	66.52 %	0.36	<0.001
B-HCG	440.07	38.9%	94.9 %	70 %	83.5 %	0.64	7.62	81.7 %	0.399	<0.001

In this study, 230 pregnant women between 24 and 37 weeks of gestation who were suspected of having fluid leakage were examined. Among these, 54 women (23.5%) showed suspicious fluid leakage, and 81 women (35.2%) had positive fern tests. Other demographic findings of the patients are presented in the table below (Table-1).

As shown in the table below, the mean age, weight, gestational age, gravidity, and parity among women with and without suspicious fluid leakage did not show a statistically significant difference (P>0.05). However, the levels of creatinine (0.77 mg/dl vs. 0.25 mg/dl) and β-hCG (572.76 mIU/ml vs. 123.57 mIU/ml) were significantly higher in women with suspicious fluid leakage compared to those without (P<0.05), (Table-2).

As shown in the table below, the mean age, weight, gestational age, gravidity, and parity among women with positive and negative fern tests did not show a statistically significant difference (P>0.05). However, the levels of creatinine (0.68 mg/dl vs. 0.20 mg/dl) and β-hCG (537.9 mIU/ml vs. 61.13 mIU/ml) were significantly higher in women with positive fern tests compared to those with negative fern tests (P<0.001), (Table-3).

The area under the ROC curve for creatinine in the diagnosis of suspected leakage was found to be 0.833, while for B-HCG it was 0.672. Therefore, the best cutoff point with the highest diagnostic accuracy for suspected leakage was 0.15 for creatinine and 440.07 for B-HCG (Table-4).

As shown in the table below, the highest sensitivity and specificity for creatinine in diagnosing suspected fluid leakage was achieved at a cutoff point of 0.15, with a sensitivity of 94.4% and a specificity of 58%. The positive predictive value was 40.8%, the negative predictive value was 97.1%, and the overall accuracy was 66.52%. The agreement rate was 0.36 (indicating poor agreement) (P<0.001). The highest sensitivity and specificity for B-HCG in detecting suspicious leakage was obtained at a cutoff point of 440.7, with sensitivity found to be 38.9% and specificity of 94.9%. The positive predictive value was 70%, the negative predictive value was 83.5%, and the overall accuracy was 81.7%. The concordance rate was 0.399 (indicating a weak agreement) with a significance level of P<0.001 (Table-5).

As obtained, the area under the ROC curve for creatinine in diagnosing a positive fern test in the studied women was 0.837, while for B-HCG it was 0.735. Therefore, the optimal cutoff point with the highest diagnostic accuracy for diagnosing a positive fern test was 0.145 for creatinine and 29.67 for B-HCG (Table-6).

As seen in the table below, the highest sensitivity and specificity for creatinine in diagnosing a positive fern test was obtained at a cutoff point of 0.145, with sensitivity at 88.9% and specificity at 64.4%. The positive predictive value was 57.6%, the negative predictive value was 91.4%, and the overall accuracy was 73.04%. The concordance rate was 0.474 (indicating moderate agreement) with a significance level of P<0.001. The highest sensitivity and specificity for B-HCG in diagnosing a positive fern test was obtained at a cutoff point of 29.67, with sensitivity at 64.2% and specificity at 73.2%. The positive predictive value was 56.5%, the negative predictive value was 79%, and the overall accuracy was 70%. The concordance rate was 0.362 (indicating weak agreement) with a significance level of P<0.001 (Table-7).

## Discussion

**Table:** Table6. Area Under the Curve (AUC) for creatinine and B-HCG in diagnosing a positive fern test in the studied women

Variable	AUC	Standard ad Error	Sig	Confidence interval
				Minimum	Maximum
Cr	0.837	0.026	0.000	0.786	0.889
B-HCG	0.735	0.035	0.000	0.667	0.804

**Table:** Table7. The sensitivity and specificity of creatinine and B-HCG in diagnosing a positive fern test in the studied women based on the optimal cutoff point

	Cut-off Point	Sensitivity	Specificity	PPV	NPV	LR-	LR+	Overall Accuracy	Kappa	Significance
Cr	0.145	88.9 %	64.4 %	57.6 %	91.4 %	0.172	2.49	73.04 %	0.474	<0.001
B-HCG	29.67	64.2	73.2 %	56.5 %	79%	0.489	2.39	70 %	0.362	<0.001

This study examined the diagnostic utility of creatinine and B-HCG in identifying fluid leakage and positive fern tests among a cohort of 230 pregnant women between 24 and 37 weeks of gestation who presented with suspected fluid leakage. The findings highlight essential differences in laboratory values related to suspected leakage and positive fern tests, emphasizing the clinical significance of these biomarkers in obstetric care. The area under the ROC curve analysis offered further insights, with the area being 0.833 for creatinine and 0.672 for B-HCG in diagnosing suspected leakage. The best cutoff points were 0.15 mg/dl for creatinine and 440.07 mIU/ml for B-HCG. These values suggest that while both markers can assist in diagnosing suspected fluid leakage, creatinine shows a superior diagnostic capability. Also, creatinine demonstrated a high sensitivity of 94.4% and a modest specificity of 58% at the cutoff point of 0.15 mg/dl. In contrast, B-HCG exhibited a significantly lower sensitivity of 38.9% but a high specificity of 94.9% at a cutoff of 440.07 mIU/ml. The high negative predictive value for creatinine (97.1%) highlights its utility in ruling out fluid leakage, whereas B-HCG’s higher positive predictive value (70%) can be critical in confirming the diagnosis when the test result is positive. The findings of this study advocate for careful consideration of creatinine and B-HCG levels when evaluating pregnant patients for suspected amniotic fluid leakage and positive fern tests. The high sensitivity of creatinine suggests that it can be an effective initial screening tool, while the specificity of B-HCG may prove useful in confirming diagnoses. Furthermore, the concordance rates highlight areas for improvement. The relatively low concordance rates (0.36 for creatinine and 0.362 for B-HCG) call for further refinement in the use of these tests to improve clinical decision-making and reduce false-positive or negative results. In the study by Jasmina Begum and colleagues [15] in 2017 on pregnant women between 28 to 42 weeks, it was found that women with PROM had significantly higher levels of vaginal urea and creatinine compared to healthy women. The results of this study are in line with our findings. In our study, it was also determined that the level of creatinine in women with suspected leakage and positive fern test was significantly higher than in women with negative results. In the study by C Gezer and colleagues [16] in 2017, which was conducted on 100 pregnant women between 24 to 37 weeks with PROM in a case-control design, it was found that elevated levels of urea and creatinine in the vaginal fluid were significantly associated with premature rupture of membranes (PPROM). The results of this study align with our findings. In our study, it was also determined that the level of creatinine in women with suspected leakage and positive fern test was significantly higher than in women with negative results. Among the research conducted to evaluate the diagnostic efficacy of creatinine in cervical-vaginal secretions for identifying PROM, the 2004 study by Gorbz and Karatek stands out. In this study, a threshold of .12 mg/dL was determined, and utilizing the ROC curve, the method achieved a sensitivity, specificity, positive predictive value, and negative predictive value of 100% for diagnosing premature rupture of fetal membranes [17]. Although in our study, the cutoff point is close to the threshold introduced in the mentioned study, the accuracy achieved in our study regarding the diagnosis of leakage through the fern test and suspected leakage is lower than in that study. This discrepancy may be attributed to differences in the sample size, demographic characteristics of the patients, the inclusion and exclusion criteria of the study, and variations in patient selection. In the study by Kafali and Oksalir in 2007, aimed at determining the diagnostic power of urea and creatinine in cervical-vaginal secretions for diagnosing premature rupture of membranes, Using the ROC curve, a cutoff point of 0.6 mg/dL for creatinine and 12 mg/dL for urea was established. The researchers reported a sensitivity, specificity, positive predictive value, and negative predictive value of 100% for urea and creatinine in cervical-vaginal secretions for diagnosing premature rupture of membranes [7]. In our study, the highest sensitivity and specificity for creatinine in diagnosing a positive fern test were found at a cutoff point of 0.145 mg/dL, with a sensitivity of 88.9% and a specificity of 64.4%. The positive predictive value was 57.6%, the negative predictive value was 91.4%, and the overall accuracy was 73.4%. The agreement rate was 0.474, indicating a moderate level of agreement (P<0.001). While the level of agreement obtained was moderate, it was not as high as reported in the aforementioned study. The findings of this study indicate that creatinine and BHCG levels are significantly elevated in women with premature rupture of membranes (PROM), with creatinine demonstrating a notably greater diagnostic power compared to BHCG. Overall, tests showed inadequate agreement with the fern test, exhibiting a moderate correlation for creatinine and a poor correlation for BHCG. Consequently, creatinine and BHCG may be utilized in conjunction with standard tests for diagnosing PROM. By employing effective diagnostic methods, it is possible to avert the potentially life-threatening complications associated with PROM, and prompt treatment can mitigate adverse outcomes. Further research involving a larger sample size and careful control of confounding variables is warranted.

## Conclusion

In summary, the differential diagnostic value of creatinine and B-HCG in identifying fluid leakage and positive fern tests among pregnant women suggests these biomarkers can play pivotal roles in obstetric care. Future studies should explore the integration of these markers into clinical protocols to enhance detection accuracy and ensure better outcomes for mothers and their infants. Additionally, awareness of the limitations of these tests and improving their predictive power could significantly advance prenatal care practices.

## Conflict of Interest

None.
